# Health anxiety, mental defeat and fear of illness recurrence and progression following a cardiac event: Implications for cardiac rehabilitation

**DOI:** 10.1111/bjhp.70068

**Published:** 2026-04-17

**Authors:** Sean Hill, Sarah Scott, Paul M. Salkovskis

**Affiliations:** ^1^ Department of Experimental Psychology University of Oxford Oxford UK; ^2^ Buckinghamshire Healthcare NHS Foundation Trust Aylesbury UK; ^3^ Oxford University Hospitals NHS Foundation Trust Oxford UK; ^4^ Oxford Health NHS Foundation Trust Oxford UK

**Keywords:** cardiac event, cardiac rehabilitation, fear of recurrence, health anxiety, mental defeat

## Abstract

**Objectives:**

To evaluate the contribution of health anxiety, mental defeat and fear of recurrence and progression (FRP) as variables in the adjustment process following cardiac events and subsequent wellbeing, adjustment and rehabilitation.

**Design:**

A two‐part study was conducted: cross‐sectionally examining psychological factors shortly following a cardiac event and longitudinally examining how these variables were associated with adherence and physical/psychological outcomes of cardiac rehabilitation.

**Methods:**

A UK‐based sample of post‐cardiac event patients (*N* = 176, *M*_age_ = 66.1, SD = 10.0) was categorized as high health anxiety with depression and/or anxiety, depression or anxiety only, or neither health anxiety nor depression/anxiety. Mental defeat and FRP were compared across groups pre‐rehabilitation and examined in relation to adherence to, and outcomes of, an 8‐session cardiac rehabilitation programme.

**Results:**

Analyses indicated significantly higher mental defeat and FRP in those with health anxiety than in the other groups. However, regression analyses showed that neither health anxiety, mental defeat, nor FRP was significantly associated with rehabilitation adherence or outcomes.

**Conclusions:**

This study identified mental defeat and FRP as important factors in health‐anxious cardiac patients, with implications for the coping and adjustment process and rehabilitative efforts. No linear association between these variables and rehabilitation adherence and outcomes was found, suggesting that more nuanced approaches to identifying their impact on rehabilitation may require development.

**What is already known?**

- Health‐related anxiety is over‐represented in individuals with cardiac conditions, although the nature of this relationship is not conclusively established.
- There is also limited information about health‐related anxieties in recent cardiac event patients.

**What does the study add?**

- This study demonstrates that health anxiety, mental defeat and fear of illness recurrence or progression (FRP) are highly prevalent in individuals who have recently experienced a cardiac event.
- This study indicates the need for further research into the impact of health‐related anxieties on coping, adjustment and rehabilitative efforts following cardiac events.

## INTRODUCTION

### Psychological burden of cardiac events

Experiencing a cardiac event, defined as events such as a myocardial infarction, cardiac arrest or other acute coronary syndrome (Lerman & Zeiher, 2005), imposes significant physical and psychological challenges. As survival rates of these events have increased due to healthcare innovations, so too has the need to understand the impairments and disabilities faced by survivors. While the medical outcomes are well documented, the psychological profile of patients remains significantly under‐researched. This is despite the fact that cardiac events are, by nature, significant adverse experiences, typically resulting in lasting changes to sense of self, health and future (Murphy et al., 2023; Randall et al., 2009).

Rates of depression and anxiety are elevated in post‐event patients than in the general medical outpatients, although reported rates vary widely, from 10% to 52% (Bunch et al., 2004; Schaaf et al., 2013). Adverse psychological impact is typically greatest during hospitalization immediately following the event, may persist in the absence of appropriate support, though it is amenable to improvement when such support is provided (Larsson et al., 2014). These associations are reflected by the requirement for routine assessment of depression and anxiety symptoms in cardiac rehabilitation (BACPR, 2023; NICE, 2015).

Post‐traumatic stress symptoms are also common, with prevalence 2.5–3 times higher in cardiac event survivors compared to other cardiovascular conditions (Agarwal et al., 2019). These symptoms, especially avoidance, psychosocial numbing, hyperarousal and perceptions of recovery (Presciutti et al., 2019), can be persistent, with 31% of survivors meeting clinical thresholds for PTSD 6 months post‐event (Presciutti et al., 2018).

Health‐related anxiety is also prevalent in cardiac populations and important in cardiovascular prognosis (Berge et al., 2016; Hohls et al., 2020). ‘Health anxiety’, or ‘hypochondriasis’, is defined by the International Classification of Diseases—ICD‐11 (6B23; World Health Organization, 2022) as a preoccupation and/or fear of experiencing a serious or life‐threatening illness. In the context of cardiac health, it is described under numerous terms, including cardiac anxiety (Rosman et al., 2015), heart‐focused anxiety (Eifert et al., 2000) and cardiophobia (Eifert, 1992). Across definitions, these anxieties may interfere with health‐restoring behaviours, such as exercise, medication adherence and dietary regulation (Bauer et al., 2012; Schmitz et al., 2022). They also often interfere with other aspects of health‐related quality of life (Smith et al., 2015), including sleep, stress management and daily functioning (Wachelder et al., 2009). Health‐related anxieties present an immediate concern in this population, as poor adherence to treatment and health behaviours are prospectively associated with further cardiac events and all‐cause mortality (Chowdhury et al., 2013).

### Cardiac rehabilitation and treatment adherence

Cardiac rehabilitation is an essential component of management following cardiac events. In the UK, structured cardiac rehabilitation programmes within National Health Service (NHS) trusts are endorsed by National Institute of Health and Care Excellence (NICE) quality standards and guidelines (NICE, 2013, 2015) and the NHS long‐term plan (NHS Digital, 2024). Programmes follow standards set by the British Association for Cardiovascular Prevention and Rehabilitation (BACPR, 2023), which include exercise training, education and psychological support. Programmes ideally commence within 2 weeks of a patient's hospital discharge (BACPR, 2023) and typically span 6–12 weeks (Buckley et al., 2013).

Adherence to all components of rehabilitation can be challenging and is influenced by numerous physical and psychological barriers (Bitton et al., 2013). Demographic inequalities also appear prominent, with lower participation and completion rates associated with younger age, minoritized ethnic group status (Zhang et al., 2017), current smoking, low socioeconomic status (Turk‐Adawi et al., 2014), obesity (Biswas et al., 2018), low health literacy, comorbid physical health conditions (Gaalema et al., 2017) and a longer delay between hospital discharge and beginning rehabilitation (Ruano‐Ravina et al., 2016). Physical barriers include access to financial resources, transportation, medication and geographical proximity to healthcare services (Erskine et al., 2018).

In contrast, psychological barriers to adherence are comparatively under‐researched. Depressive symptoms, health‐related anxiety and maladaptive health behaviours can influence healthcare utilization (Hohls et al., 2020), quality of life and cardiovascular prognosis (Nakamura et al., 2013; Tully et al., 2016). However, these relationships can be complex and may only be detectable in large samples. For instance, Hohls et al. (2020) found that ‘avoidance’ and ‘attention’ components of cardiac anxiety, but not ‘fear’, predicted healthcare utilization, though the effect sizes were modest (*OR* = 0.69–1.76) and emerged only in a very large sample (*n* > 1000). Therefore, while psychological variables like cardiac anxiety are relevant to healthcare utilization and adherence, the nature and mechanisms of these relationships are not yet well established.

### Mechanisms linking cardiac recovery and psychological factors

Transient periods of health anxiety are common, and to an extent expected, following serious health events (Lebel et al., 2020). For some, this develops into more sustained anxiety, resulting in prolonged distress and functional impairment (Bauer et al., 2012). Health anxiety has been linked to reduced treatment adherence across a range of physical health conditions (Rode et al., 2006; Tang et al., 2007), is overrepresented in cardiac patients (Berge et al., 2016) and is associated with increased risk of recurrent cardiac events and poor prognosis (Tully et al., 2016).

As such, examining the nature and trajectory of health anxiety in patients recovering from recent cardiac events is essential to mitigating disease burden. To our knowledge, no research has examined the incidence or course of health anxiety specifically in patients following a cardiac event. This is despite cardiovascular disease and its associated risk factors being among the physical health conditions most strongly linked to health anxiety, akin to cancer and diabetes (Norbye et al., 2022). This population is of particular interest considering cardiovascular rehabilitation is routinely commenced shortly after patients' index events (BACPR, 2023; Gaalema et al., 2017), wherein transient health anxiety may be more pronounced.

The cognitive‐behavioural model of health anxiety (Salkovskis et al., 2003) offers a useful framework for examining these processes. It proposes individuals' reactions to health‐related information are shaped by pre‐existing beliefs about health and illness. ‘Maladaptive’ assumptions about health can lead to catastrophic interpretations of bodily symptoms or medical advice, reinforcing anxiety and unhelpful coping behaviours.

The relationship between health anxiety and both the adjustment to cardiac events, and subsequent rehabilitation engagement, is likely nuanced and possibly non‐linear. Whereas high levels of health anxiety could reduce engagement through excessive worry, avoidance or catastrophic beliefs (Salkovskis et al., 2003), low levels of health anxiety could also reasonably reduce engagement with healthcare (e.g., due to lack of concern). Alongside this, understanding how symptomology affects prognosis is equally important. Without this, these associations may appear counterintuitive, as health‐related vigilance, checking behaviours and avoidance behaviours may be feasibly considered to be health‐protective. The proposed model (Salkovskis et al., 2003) instead argues that when these behaviours are driven by psychological distress, these may (1) provide no additional benefit to patient's health than routine medical care; (2) inadvertently worsen quality of life through restriction of meaningful activity. Although physiological variables such as chronic stress, allostatic load, oxidative stress, autonomic dysregulation and immune dysfunction have been identified as a potential biological mechanism linking anxiety and poorer health (Costa et al., 2022; Felix et al., 2023), it is important to consider behavioural mechanisms, given their relevance to rehabilitation adherence and day‐to‐day functioning.

In medical contexts, the health anxiety model may benefit from integrating related psychological constructs to better capture the complexity of health‐related distress (Salkovskis et al., 2016). Although elevated health anxiety is associated with lower treatment adherence in some chronic illness groups (Wang et al., 2025), these findings do not yet clarify whether reducing health anxiety leads to improved engagement or health behaviours, and also remain unexplored in cardiac populations, wherein effective rehabilitation is critical to recovery.

Accordingly, we propose two constructs may be especially relevant to consider for post‐event patients when considering implications for cardiac rehabilitation. These are: (1) mental defeat; (2) fear of illness recurrence and progression (FRP). Both constructs have been conceptualized as potentially adaptive or maladaptive responses to physical illness, depending on their presentation (Coutts‐Bain et al., 2026; Tang et al., 2010), and offer promise for incorporating into psychological treatments, despite currently limited application (Coutts‐Bain et al., 2026; Murata et al., 2019).

### Mental defeat

Mental defeat is a psychological state wherein one's circumstances lead to persistent feelings of powerlessness, loss of agency, demoralization and being overwhelmed (e.g., Tang et al., 2007). It is thought to emerge when past experiences, particularly those involving perceived failure or helplessness, are activated by current adverse or life‐altering circumstances (Ehlers et al., 1998). Mental defeat can be understood as a form of catastrophising regarding social and role functioning, sense of identity and social status in response to significant changes, which may include acute or chronic physical illness. Anecdotally, a cardiac patient may exhibit mental defeat through catastrophising thoughts such as: ‘These symptoms mean I am useless to my family and friends’, or ‘I'll never be the person I once was’. These thoughts can reinforce cycles of negative thinking, hopelessness and self‐criticism which may reduce motivation to engage in rehabilitation.

By inhibiting productive action and goal‐directed behaviour, mental defeat has been proposed as a mechanism underlying poor treatment response in the context of both traumatic events (Ehlers et al., 1998) and depression (Gilbert & Allan, 1998). This suggests potential relevance for cardiac event patients, whose recovery typically involves adapting to sudden, life‐altering circumstances.

### Fear of illness recurrence and progression

By contrast to mental defeat, fear of illness recurrence focuses exclusively on future‐oriented concerns about the return of illness, while fear of illness progression involves concerns that the condition will worsen in severity or impact (e.g., Bergerot et al., 2022). These concepts were primarily developed within oncology (Crist & Grunfeld, 2013), but more recently regarded as important across a range of physical conditions (Sharpe et al., 2024). Due to their conceptual similarities, recent work has proposed a unified framework encapsulating fear of illness recurrence and progression (FRP; Coutts‐Bain et al., 2022; Sharpe et al., 2023).

FRP represents a specific type of health anxiety relevant to those living with or recovering from serious physical illness. It involves overestimation of the likelihood of event recurrence and progression, which motivates health anxiety‐related safety‐seeking behaviours such as excessive symptom monitoring, catastrophising, avoidance and rumination (Salkovskis et al., 2003). Emerging evidence suggests FRP is highly prevalent among cardiac patients and associated significantly with cardiac anxiety (Clarke et al., 2024; Sharpe et al., 2024). Severity of recurrence fears appear greater shortly after onset of a health event (Fait et al., 2018) and associated with negative coping styles in cardiac patients (Zhen et al., 2022), suggesting high relevance to the context of post‐cardiac event patients.

### The present study

Within a cognitive‐behavioural health anxiety framework, FRP may interact with mental defeat through shared catastrophising beliefs about illness worsening and impact of the illness on identity, status and social roles. This may offer a mechanistic explanation predicting poorer adherence to medical treatments and lifestyle modifications among cardiac patients with elevated health anxiety (Berge et al., 2016; Petricone‐Westwood et al., 2019).

Taken together, health anxiety, mental defeat and FRP could account for variance in both physical and psychological outcomes following a cardiac event. The present study seeks to evaluate this in the context of cardiac rehabilitation. Post‐event patients will be divided into those with high levels of psychological distress (either with or without clinically elevated health anxiety) and those without evident psychological distress.

The primary hypothesis is that cardiac patients with elevated health anxiety and anxiety or depression will report significantly higher scores on measures of both mental defeat and FRP relative to patients with depression and/or anxiety alone and patients without depression or anxiety. The secondary hypothesis is that health anxiety, mental defeat and FRP will each be uniquely associated to (a) poorer completion rates of cardiac rehabilitation and (b) poorer psychological and physical improvements following rehabilitation.

## METHOD

Ethical approval for this study was obtained following a favourable opinion from the Tyne and Wear South Research Ethics Committee (24/NE/0041). The study was sponsored by University of Oxford Research Governance, Ethics & Assurance (RGEA, PID17286‐SI001) and approved via the Integrated Research Application System (IRAS ID 331618). This study was not pre‐registered prior to commencement.

### Participants and sampling procedure

The study involved three groups, comprising people who experienced a recent cardiac event with: (1) health anxiety and at least one of anxiety or depression (‘HA + AD’); (2) at least one of depression or anxiety, and not health anxiety (‘AD‐only’); (3) none of depression, anxiety or health anxiety (‘non‐clinical’). This three‐group categorization was chosen to isolate the potential additive or unique contribution of health anxiety relative to general anxiety and depression. This approach was preferred to alternative (e.g., correlation matrices), as it preserved clinically meaningful groupings and avoided obscuring the shared variance in comorbid presentations. Participants with health anxiety only, without comorbid anxiety or depression, were rare (*n* = 17, 9.7%, Primary hypothesis section) and excluded from group comparisons to maintain clear and interpretable diagnostic groupings.

Eligibility criteria were (1) age 18+, (2) competency in English and (3) eligibility for cardiovascular rehabilitation at the research site, with no exclusion criteria. Potential participants were recruited from an NHS hospital containing a cardiac rehabilitation team in Oxford, England. They were invited to participate by hospital staff during in‐person assessments, provided routinely to patients following their cardiac event prior to rehabilitation.

### Study design

Data were collected from two time points: (1) shortly after their cardiac event, before starting cardiovascular rehabilitation; (2) following completion of an 8‐session cardiovascular rehabilitation programme.

There were two components to the study: (1) a cross‐sectional between‐subjects design at time point one; (2) a prospective cohort regression design at both time points. The cross‐sectional aspect focused on main differences between participants' responses on measures of psychological variables at the point of contact by health professionals following a cardiac event. The regression aspect focused on differences between participants' responses on measures of psychological variables to cardiovascular rehabilitation outcomes.

### Measures

- The clinical demographics collected included participants’ age, gender, ethnic, marital status, pre‐rehabilitation smoking status, BMI and days between hospital admission and contact with the rehabilitation team.
- The Hospital Anxiety and Depression Scale (HADS) is a 14‐item assessment of anxiety and depression symptoms in hospital settings, using 1–4 Likert‐scales with higher scores indicating greater severity. The scale shows strong reliability for depression (α = .93) and anxiety (α = .90), (Norton et al., 2013) and good convergent and concurrent validity (Bjelland et al., 2002). The HADS also performs well in medical populations diagnostically, with the recommended cut‐off of ≥8 on both the HADS‐A and HADS‐D subscales typically yielding sensitivity and specificity of approximately 0.80 for identifying anxiety and depressive disorders (Bjelland et al., 2002). A cut‐off of ≥11 is more strongly supported for research purposes (Brennan et al., 2010), and so was adopted for grouping purposes in this study. In the current sample, internal consistency was excellent for the anxiety (α = .88) and depression (α = .88) subscales.
- The Short Health Anxiety Inventory (SHAI) is a 14‐item measure of health anxiety symptoms using 1–4 Likert‐scale questions, with higher scores indicating greater health anxiety. It shows convergent, discriminant and criterion‐related validity (Salkovskis et al., 2002) and strong internal consistency (α = .85–.94; Alberts et al., 2013). Regarding diagnostic performance, the SHAI shows excellent discriminatory ability: a cut‐off of 22 yields 93% sensitivity and 85% specificity in psychiatric samples (Alberts et al., 2013), while lower cut‐offs (e.g., 13–18) are suitable for screening in community or mixed medical samples (Österman et al., 2022). Consistent with NHS IAPT screening guidance (NHS England, 2017), which adopts a ≥14 threshold for identifying potential cases of health anxiety, this study used the same cut‐off to maximize sensitivity during initial classification. Internal consistency in this sample was α = .90.
- The Mental Defeat Self Perception Questionnaire (MDSP) is a 20‐item measure of mental defeat which uses Likert scales ranging 1–5, with higher scores indicating greater mental defeat. Psychometric validation is limited, but construct validity and internal consistency can be inferred through empirical studies using it alongside other relevant measures (e.g., Murata et al., 2019; Nagata et al., 2018). In the present study, internal consistency was α = .96.
- The Worry about Recurrence or Progression Scale (WARPS) is an 18‐item measure of FRP, using 1–5 Likert‐scales ranging from ‘strongly disagree’ to ‘strongly agree’, with higher scores indicating greater FRP. The WARPS shows good construct validity, excellent internal consistency (α = .96) and test–retest reliability (Sharpe et al., 2024). In the current sample, internal consistency was α = .96.
- Physical fitness was measured using a Metabolic Equivalent Level (MET), which measures ‘fitness’ level using maximal output from a bike test, completed at the first and last rehabilitation sessions.
- The Dartmouth Quality of Life Index (DQOL) is a 9‐item measure of health‐related quality of life using 1–5 Likert scales, with higher scores indicating lower quality of life. The scale has evidenced discriminant and convergent validity (Gherunpong et al., 2004). In the current sample, internal consistency was good (α = .85).

### Procedure

Participants were recruited between 25 August 2024 and 31 April 2025. A total of 249 eligible patients were approached, of whom 178 (71.5%) consented to participate (71 declined, 28.5%). Those who opted in were emailed a link to an online questionnaire, which contained an information sheet, psychometric measures (SHAI, MDSP, WARPS) and consent form for researchers to access their hospital data after they complete rehabilitation. Using pseudonymized codes, the routinely collected pre‐rehabilitation data (HADS, DQOL and baseline clinical demographics) were matched to participants' online questionnaire data.

Participants were provided an eight‐session cardiac rehabilitation programme compliant with BACPR (2023) standards. At the first session, MET was recorded to measure baseline fitness. This was repeated at the final session, followed by measuring HADS, DQOL and BMI. Whether participants completed their programme or self‐discharged early was also recorded.

### Data analysis

#### Data processing

Adherence data were originally extracted as the number of rehabilitation sessions attended, with the intention of analysing adherence as a continuous variable. However, inspection of the data showed that participation patterns were highly polarized, with only 5 of 163 main sample participants (≈3%) beginning rehabilitation but not completing it, while the remainder either attended all sessions or declined to start. This resulted in a strongly bimodal, non‐normal and heteroscedastic distribution, violating the assumptions of linear modelling (Tripepi et al., 2008) and leaving a continuous measure of adherence conceptually uninformative. To preserve statistical validity and interpretability, programme adherence was operationalized as a binary variable: participants who completed rehabilitation were classified as ‘adherent,’ and those who declined or did not complete were classified as ‘non‐adherent.’ Participants who were not offered rehabilitation (typically due to being medically unfit, out‐of‐area or having another health event) were not included in adherence analysis.

#### Statistical analysis

To test the primary hypothesis, dependent variables (MDSQ, WARPS) were compared between groups using analysis of variance (ANOVA). Participants who scored above threshold for health anxiety (SHAI ≥ 14) but below threshold for depression and anxiety (HADS‐A < 11; HADS‐D < 11) were excluded from group comparisons to ensure clear diagnostic categorization. Where ANOVA indicated a significant main effect of group, post‐hoc comparisons were conducted using Tukey's HSD when equal variances were assumed and Dunnett's T3 when not assumed. Equality of variances was assessed using Levene's test.

To test the secondary hypothesis (a), whether health anxiety, mental defeat and FRP predicted treatment adherence, a binary logistic regression was conducted with rehabilitation completion (adherent vs. non‐adherent) as the binary outcome variable. Age, sex, days between hospital admission and commencing rehabilitation, and baseline depression and anxiety were considered as potential covariates but not included in the final model as none were significantly associated with adherence.

To test secondary hypothesis (b), whether health anxiety, mental defeat and fear of FRP predicted treatment outcomes, multiple linear regressions examined whether health anxiety, mental defeat and FRP uniquely predicted change in each outcome. To reduce the risk of Type I error and maintain statistical power, only one psychological outcome (quality of life) and physiological outcome (fitness level) were selected for regression analysis. Quality of life was selected over other candidates (anxiety and depression) as it offers a comprehensive indication of psychological and functional adjustment; fitness level was chosen (over change in BMI), as higher scores provide an unambiguous indicator of improvement, independent of premorbid weight. To account for baseline differences, both regressions were repeated using residual change scores (post‐rehabilitation scores regressed on pre‐rehabilitation scores for DQOL and MET). As anticipated, there was a small group of participants who scored above threshold on health anxiety but not for either depression or anxiety (*n* = 17). As an exploratory analysis, the ANOVAs for mental defeat and FRP were carried out across the four groups.

Effect sizes are reported as Cohen's *d* for mean differences, η^2^ or partial η^2^ for ANOVAs and Cramér's V for chi‐square analyses, consistent with conventional standards (Cohen, 1992; Rosnow & Rosenthal, 2003).

#### Power analysis

A meta‐analysis by Easton et al. (2016) explored anxiety prevalence among heart failure patients, reporting 28.79% for clinical levels of anxiety and 55.46% for elevated anxiety. The prevalence rate of all anxiety disorders in the UK general population is 10.2% (NHS Digital, 2016), which provides an odds ratio of 3.528 compared to cardiac patients, giving a Cohen's‐*F* value of 0.348 which falls in the medium‐large effect size range; with 95% power at an α level of 0.05, a sample size of 132 participants (44 per group) will be required. To account for calculation imprecision, 150 participants (50 per group) were the target sample size.

To assess this study's power to detect treatment adherence effects, a post‐hoc power analysis using the odds ratio reported by Hohls et al. (2020) for the relationship between cardiac anxiety and exercise participation (OR = 1.76) indicated a sample of ~504 participants would be required to achieve 80% power to detect an effect of this magnitude for a dichotomous participation outcome (α = .05). Given our analytic sample (*N* ≈ 150), the study is underpowered to reliably detect small‐to‐moderate effects on adherence: findings related to rehabilitation adherence and outcomes are therefore planned and interpreted as exploratory.

## RESULTS

### Participants

**FIGURE 1:**
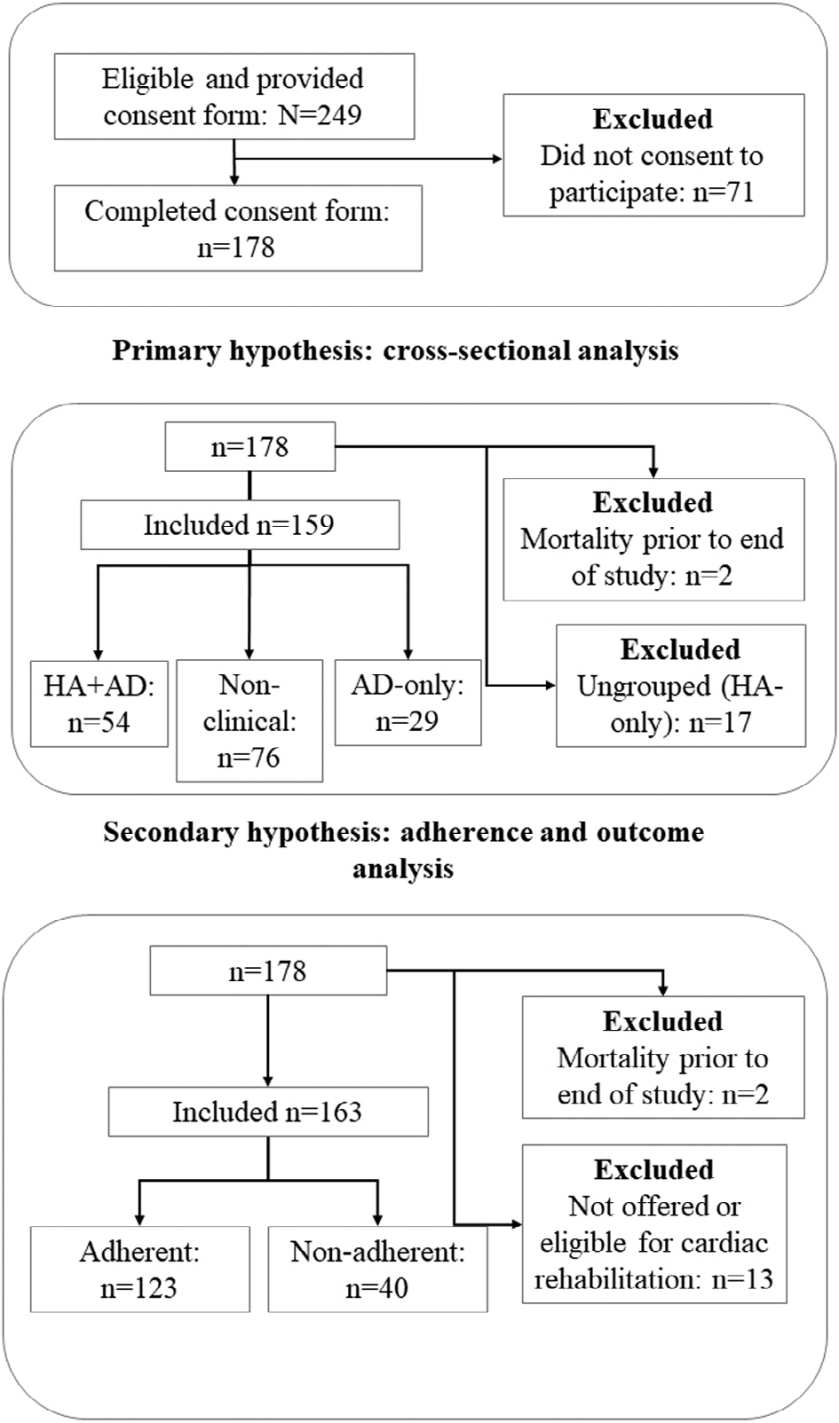
CONSORT‐style flow diagram of participant recruitment and categorization (AD, anxiety and/or depression; HA, health anxiety). Ungrouped is HA caseness only.

Of the 249 eligible participants approached, a total of 178 patients completed the online forms; two were excluded due to recorded mortality, providing a final sample of 176. A CONSORT‐style flow diagram is presented in Figure 1, and full details in the supplementary document. The sample comprised 130 males (73.9%) and 46 females (26.1%), aged 37.2–90.1 years (*M* = 66.1, SD = 10.0). The time between participants' cardiac event and initial contact from the cardiac rehabilitation team ranged 6–159 days (*M* = 48.2, SD = 24.7).

Regarding smoking status, 28 (15.9%) identified as current smokers and 148 (84.1%) were non‐smokers. For marital status, 136 (77.3%) were married, 11 (6.3%) partnered, five (2.8%) separated, nine (5.1%) widowed and 15 (8.5%) single. For occupational status, 73 (41.5%) were retired, 51 (29.0%) employed full‐time, 27 (15.3%) employed part‐time, four (2.3%) unemployed and one (0.6%) student. For ethnicity, 130 (73.9%) identified as White British, 20 (11.4%) as White Other, 13 (7.4%) as Asian Other, five (2.8%) as Asian Mixed and four (2.3%) as Black African. Ethnicity was not stated by four participants (2.3%).

### Primary hypothesis

In testing the primary hypothesis, 17 participants (9.7%) were excluded due to scoring above threshold for health anxiety and not depression or anxiety, providing a main sample of 159. This comprised 54 in the ‘health anxiety and depression/anxiety’ (HA + AD) group, 29 in the ‘depression/anxiety only’ (AD‐only) group and 76 in the ‘non‐clinical’ group. Sample demographics are presented in Table 1.

**TABLE 1:** Main sample demographics.

Measure	HA + AD group (*n* = 54)	AD‐only group (*n* = 29)	Non‐clinical group (*n* = 76)	Between‐groups comparative statistics^1^
Gender: *n* (%)				
Female	22 (40.7)	9 (31.0)	11 (14.5)	***χ*** ^2^_**(2, *N* = 159)**_ = 11.60, *p* = .003*; Cramer's *V* = .27
Male	32 (59.3)	20 (69.0)	65 (85.5)	
Ethnicity: *n* (%)				
White British	37 (71.2)	21 (72.4)	60 (80.0)	*χ* ^2^_(2, *N* = 156)_ = 1.51, *p* = .471; Cramer's *V* = .10
White other	7 (13.5)	4 (13.8)	7 (9.3)	
Asian (Mixed or Other)	7 (13.5)	4 (13.8)	1 (1.3)	
Black African	0 (0.0)	0 (0.0)	3 (4.0)	
Other mixed	1 (1.9)	0 (0.0)	4 (5.3)	
Employment status: *n* (%)				
Retired	22 (40.7)	10 (34.5)	31 (40.8)	*χ* ^2^_(4, *N* = 159)_ = 2.46, *p* = .652; Cramer's *V* = .09
Full‐time employed	15 (27.8)	12 (41.4)	21 (27.6)	
Part‐time employed	17 (31.5)	6 (20.7)	21 (27.6)	
Unemployed	0 (0.0)	1 (3.4)	2 (2.6)	
Student	0 (0.0)	0 (0.0)	1 (1.3)	
Marital status *n* (%)				
Married	44 (81.5)	23 (79.3)	56 (73.7)	*χ* ^2^_(2, *N* = 159)_ = 1.74, *p* = .419; Cramer's *V* = .11
Partnered	4 (7.4)	1 (3.4)	5 (6.6)	
Separated	1 (1.9)	2 (6.9)	2 (2.6)	
Widowed	2 (3.7)	1 (3.4)	5 (6.6)	
Single	3 (5.6)	2 (6.9)	8 (10.5)	
Smoking status *n* (%)				
Smoker	6 (11.1)	6 (20.7)	14 (18.4)	*χ* ^2^_(2, *N* = 159)_ = 1.72, *p* = .450; Cramer's *V* = .10
Non‐smoker	48 (88.9)	23 (79.3)	62 (81.6)	
Age: M (SD)	64.9 (10.0)	66.2 (7.5)	65.8 (10.6)	*F*_(2,156)_ = 0.20, *p* = .820, *η* ^2^ = .00
BMI: M (SD)	28.1 (4.0)	29.5 (5.1)	27.7 (4.2)	*F*_(2,155)_ = 1.94, *p* = .152, *η* ^2^ = .02
Days from event to contact: M (SD)	52.7 (26.4)	42.7 (26.8)	46.1 (21.6)	*F*_(2,156)_ = 1.93, *p* = .150, *η* ^2^ = .02
Baseline depression^2^	9.7^a^ (4.0)	8.2^a^ (3.8)	3.0^b^ (2.8)	***F***_**(2,156)**_ **= 64.67, *p* < .001** * **, *η*** ^**2**^ **= .45**
Baseline anxiety^2^	10.8^a^ (3.4)	11.7^a^ (3.3)	3.3^b^ (2.9)	***F***_**(2,156)**_ **= 122.34, *p* < .001** * **η** ^**2**^ **= .61**
Baseline health anxiety^2^	19.5^a^ (4.2)	7.0^b^ (4.3)	7.4^b^ (3.1)	***F***_**(2,156)**_ **= 189.94, *p* < .001** * **η** ^**2**^ **= .71**
Baseline quality of life^2^	20.4^a^ (5.7)	21.6^a^ (6.3)	17.1^b^ (6.1)	***F***_**(2,154)**_ **= 7.94, *p* < .001** * **η** ^**2**^ **= .09**

^1^ For comparative analyses, employment, marital and ethnic status categories were collapsed due to small subgroup sizes. For employment, ‘full‐time’ and ‘student’ were combined, ‘part‐time’ was not combined, and ‘retired’ and ‘unemployed’ were combined to provide ‘full‐time, part‐time and not working’. For marital status, ‘married’ and ‘partnered’ were combined, as were ‘single,’ ‘widowed,’ and ‘separated,’ to provide ‘partnered or not partnered’. Ethnic status was combined to ‘White British’ and all others to provide ‘Not White British’. These combinations were made to ensure sufficient cell counts for valid statistical comparison.

^2^ Figures sharing a superscript within the same row denote that those groups did not significantly differ. For example, all figures with the superscript ‘a’ in one row did not significantly differ, but did significantly differ from figures with superscript ‘b’ in the same row.

* Statistically significant at least the *a* = .05 level, highlighted in bold for legibility.

^*^ Statistically significant at least the a = .05 level, highlighted in bold for legibility.

The groups differed significantly in terms of gender distribution, with females overrepresented in the HA + AD group and males overrepresented in the non‐clinical group, with a small‐medium effect size. Partitioned chi‐square analyses showed that this overall difference was driven by a significant difference between the HA + AD and non‐clinical groups. The groups did not significantly differ by any other demographics.

It was hypothesized that the HA + AD group will have higher mental defeat and FRP than the AD‐only and non‐clinical groups. This hypothesis was upheld for both mental defeat and FRP. For mental defeat, there was a significant main effect of group (*F*_(2,156)_ = 9.07, *p* < .001) with a medium‐large effect size (Cohen's *d* ≈ 0.76). As variances were unequal, (*F*_(2,156)_ = 5.94, *p* = .003), Dunnett's T3 was used for post‐hoc comparison. The HA + AD group reported significantly higher scores than the AD‐only group (*p* = .048) and non‐clinical group (*p* < .001). No significant difference was found between the AD‐only and non‐clinical groups.

For FRP, there was a significant main effect of group (*F*_(2,156)_ = 16.78, *p* < .001) with a large effect size (Cohen's *d* ≈ 1.05). Tukey HSD was selected for post‐hoc comparison as no violations of parametric testing were found. This analysis indicated the HA + AD group scored significantly higher than the AD‐only group (*p* = .016) and non‐clinical group (*p* < .001). As with mental defeat, no significant difference was found between the AD‐only and non‐clinical groups.

### Secondary hypothesis: Adherence

Of the total sample (*N* = 176), 13 were excluded due to not being offered rehabilitation, providing a main sample of 163. Consistent with the power analysis (Power analysis section), the sample was underpowered, indicating a high likelihood of Type II error. Of the sample, 123 (75.5%) were categorized as ‘adherent’ and the remainder (*n* = 40, 24.5%) ‘non‐adherent’. When analysed with binary logistic regression, the overall model was not statistically significant, *χ* ^2^_(3, *N* = 163)_ = 2.89, *p* = .409, and explained 2.6% of the variance in adherence (Nagelkerke *R* ^2^ = .026). None of the individual predictors were significant; further details are provided in the supplementary document. As such, neither health anxiety, mental defeat nor FRP were uniquely or together associated with cardiac rehabilitation adherence, although we reiterate cautious interpretation given the limited power of this analysis.

#### Secondary hypothesis: Treatment outcomes by group

Given the outcomes of the primary hypothesis showed the HA + AD group presented with significantly higher levels of mental defeat and FRP than other groups, it was important to assess whether these psychological profiles were associated with differential responses to rehabilitation outcomes. To assess differences in rehabilitation outcomes, mixed model (repeated measures) ANOVAs were conducted, with psychological group membership (HA + AD, AD‐only, non‐clinical) as the grouping variable and pre‐ and post‐rehabilitation scores as repeated measures. This was done for fitness and quality of life. Across the main sample, there were significant improvements in both fitness and quality of life (both *p* < .001) with large effect sizes (partial *η* ^2^ *= .291* for quality of life, partial *η* ^2^ = .330 for fitness), indicating that the rehabilitation programme was broadly effective. There was also a significant time‐by‐group interaction observed for both fitness (*F*_(2,107)_ = 3.63, *p* = .030, *η* ^2^ = .064) and quality of life (*F*_(2,107)_ = 4.39, *p* = .015, *η* ^2^ = .076), indicating improvement differed between groups. Post‐hoc comparisons showed this was driven by poorer quality of life improvements in both the HA + AD and AD‐only groups relative to the non‐clinical group and poorer fitness improvements in the HA + AD group compared to the other two groups. Full statistics are reported in the supplementary document.

#### Secondary hypothesis: Predictors of treatment outcomes

Having established the presence of improvements in fitness and quality of life following rehabilitation, multiple linear regressions examined whether health anxiety, mental defeat and FRP uniquely or together predicted improvement. For fitness, the overall model was not significant, *F*_(3,106)_ = 1.50, *p* = .218, explaining 4.1% of the variance (*R* ^2^ = .041), and none of the individual predictors were significant. Similarly, for quality of life, the overall model was not significant (*F*_(3,106)_ = 0.59, *p* = .626, *R* ^2^ = .016), with no individual predictors reaching significance.

When tested again using residual scores to adjust for baseline differences, the models remained non‐significant for both fitness (*F*_(3,106)_ = 1.33, *p* = .267, *R* ^2^ = .036) and quality of life (*F*_(3,106)_ = 0.979, *p* = .405, *R* ^2^ = .027). Together, these findings indicate that neither health anxiety, mental defeat, nor FRP were uniquely or jointly associated with change in fitness or quality of life following cardiac rehabilitation.

### Exploratory analysis of four groups

Given the presence of a small group of participants reporting high levels of health anxiety without depression or anxiety (*n* = 17), exploratory ANOVAs for mental defeat and FRP were carried out. Overall, the main effects remained; for mental defeat, the HA‐only group was not significantly different from any other groups, although there was a trend relative to the non‐clinical group (*p* = 0.084). For FRP, this group was not different from the HA + AD group and was significantly different from the other two groups. Statistics for all measures are displayed in Table 2.

**TABLE 2:** Means and standard deviations of all pre‐ and post‐rehabilitation measures.

Measure	HA + AD group	AD‐only group	Non‐clinical group	Ungrouped (HA‐only)	Full sample
SHAI^a^	19.5 (4.2)	7.0 (4.3)	7.4 (3.1)	19.5 (4.9)	12.2 (7.1)
MDSP^a^	37.6 (14.7)	30.8 (13.5)	28.3 (10.0)	40.9 (18.9)	32.8 (13.9)
WARPS^a^	58.5 (14.2)	48.2 (16.1)	43.5 (14.3)	61.9 (12.5)	50.7 (16.1)
DQoL					
Pre‐rehab^a^	20.4 (5.7)	21.6 (6.3)	17.1 (6.1)	18.7 (3.9)	19.0 (6.0)
Post‐rehab^b^	19.1 (3.8)	17.2 (4.6)	15.8 (4.9)	16.2 (4.5)	17.0 (4.7)
HADS‐A					
Pre‐rehab^a^	10.8 (3.4)	11.7 (3.3)	3.3 (2.9)	3.0 (2.4)	7.0 (5.0)
Post‐rehab^b^	6.6 (3.8)	8.3 (3.4)	2.5 (2.6)	2.1 (3.4)	4.4 (4.0)
HADS‐D					
Pre‐rehab^a^	9.7 (4.0)	8.2 (3.8)	3.0 (2.8)	2.2 (2.0)	5.8 (4.6)
Post‐rehab^b^	6.0 (4.2)	5.2 (3.1)	1.9 (2.4)	1.6 (2.4)	3.5 (3.6)
MET					
Pre‐rehab^a^	4.9 (0.9)	4.7 (0.7)	4.9 (1.0)	5.2 (1.0)	4.9 (0.9)
Post‐rehab^b^	5.4 (1.1)	5.6 (1.1)	5.9 (1.4)	5.8 (1.0)	5.7 (1.3)
BMI					
Pre‐rehab^a^	28.1 (4.0)	29.5 (5.1)	27.7 (4.2)	27.4 (4.7)	28.1 (4.4)
Post‐rehab^b^	28.0 (4.3)	29.2 (4.2)	27.2 (4.1)	27.0 (4.5)	27.7 (4.2)

^a^ Sample size (*n*) for HA + AD = 54, AD‐only = 29, non‐clinical = 76, ungrouped = 17, full sample = 176.

^b^ Sample size (*n*) for HA + AD = 33, AD‐only = 18, non‐clinical = 59, ungrouped = 13, full sample = 123.

## DISCUSSION

The present study investigated the extent to which mental defeat and fear of recurrence and progression (FRP) are present in post‐event cardiac patients, whether these are more prevalent in those with health anxiety and implications for rehabilitation adherence and outcomes. We hypothesized that patients with elevated health anxiety and anxiety or depression would score significantly higher on measures of both mental defeat and FRP relative to patients with depression and/or anxiety alone and patients without depression or anxiety. This was supported, with a medium‐large effect size for mental defeat and a large effect size for FRP. Females were significantly more likely than males to experience health anxiety alongside depression and/or anxiety, and males significantly more likely to experience neither health anxiety nor depression/anxiety. Secondly, we hypothesized that health anxiety, mental defeat and FRP would be uniquely associated with poorer adherence, and psychological and physical improvements following rehabilitation. However, no evidence was found to support this, although this aspect of the study was underpowered to detect small‐to‐moderate effects, so these null findings should be interpreted as preliminary and hypothesis‐generating.

These findings are consistent with previous research demonstrating elevated health anxiety and related constructs across health and adjustment contexts (Hayter et al., 2016). While mental defeat has been studied more in trauma and pain populations (Tang et al., 2007, 2009), its presence in this sample highlights the perceived loss of agency cardiac events can evoke. Similarly, the concept of FRP, traditionally applied in oncology but more recently to other contexts (Sharpe et al., 2024), appears transferable to post‐cardiac event patients, where recurrence fears and uncertainty are also prominent (Shah et al., 2020). Our gender‐related findings are also consistent with oncology literature, wherein females consistently report higher fear of recurrence than males (Pang & Humphris, 2021).

Although mental defeat and FRP were elevated in the health anxiety group, they were not directly associated with rehabilitation outcomes, consistent with literature showing psychological predictors of improvement are often mixed and non‐linear (Yang et al., 2024). However, individuals with anxiety or depression (whether with or without health anxiety) showed less improvement in quality of life than the non‐clinical group. Moreover, those with both health anxiety and either anxiety/depression showed less improvement in fitness than both other groups. Together, this suggests the presence of comorbid anxiety or depression alongside health anxiety may have a compounded negative effect on rehabilitation outcomes, although the nuanced nature of these effects may not be fully captured within the constraints of linear analysis. Clinicians and future researchers may wish to consider longitudinal or person‐centred approaches to clarify how different psychological profiles differ in their recovery trajectories.

### Limitations

These findings should be interpreted considering several limitations. First, the outcome measures used to assess adherence, physical improvement and quality of life were relatively crude in their sensitivity and granularity. For example, adherence was measured as a binary variable out of necessity, limiting sensitivity to partial engagement or varying levels of participation. This limitation is particularly relevant as previous research has demonstrated a relatively large sample size is required to detect the nuances of adherence effects in healthcare utilization (Hohls et al., 2020), and based on this paper our sample was relatively underpowered to detect these effects with confidence. Fitness was assessed using maximal‐effort cycle tests, which may be influenced by motivation, external circumstances or variability in testing conditions. Similarly, while quality of life offers a broad indicator of psychological and functional adjustment, it may obscure more nuanced psychological changes and can be influenced by static or non‐cardiac‐related factors outside the scope of rehabilitation. However, these measures are standard clinical tools routinely used in BACPR‐compliant settings, enhancing ecological validity and translational value of findings.

Secondly, the sample was recruited from a single UK‐based cardiac rehabilitation service, which, although typical and compliant with routine UK‐based practice, limits generalizability in the international context. Thirdly, although the timing of the measures was similar across participants, individuals may follow different psychological trajectories after a cardiac event, with distress emerging at different stages of adjustment. Future research could explore these temporal dynamics using more frequent time points or qualitatively examine how symptoms differentially impact quality of life and rehabilitation efforts. Lastly, although residual change models were used to account for baseline variability, the low variance explained and non‐significant findings suggest the study may have been underpowered to detect subtle associations, and the risk of Type II error should be considered. Given the exploratory nature of our adherence and outcome analyses, future research could also consider Bayesian approaches to formally evaluate evidence for null or alternative hypotheses, which may help clarify subtle associations that frequentist methods may miss.

This study was not publicly pre‐registered, which in research can limit the transparency of analytic and methodological decisions. However, a detailed research proposal reflecting the design and analytic strategy was approved by Oxford Institute of Clinical Psychology Training and Research. This is of pertinence in this study, as it transpired that some unexpected analytic choices were required after data inspection (particularly the grouping and analysis of rehabilitation adherence and outcomes, e.g., due to unexpectedly high levels of adherence). With these limitations now salient, we emphasize future studies in this area would benefit from preregistration to enhance their interpretive confidence.

### Implications

Our findings contribute to the theoretical understanding of psychological adjustment following a cardiac event by highlighting the potential role of transdiagnostic processes such as health anxiety, mental defeat and FRP, which remain largely unexplored in this population. Individuals varied meaningfully in the degree to which they experience these processes; although unexpectedly, these were not linearly associated with rehabilitation outcomes. Although health anxiety, mental defeat and FRP did not show linear associations with rehabilitation outcomes, they may still have a meaningful role in recovery.

While this study was not designed to investigate gender as a primary variable, the overrepresentation of females in the health anxiety group warrants further exploration. Gendered experiences of cardiac rehabilitation referral, participation and programme delivery (Smith et al., 2022), as well as systemic gender and cultural disparities in help‐seeking behaviour and self‐stigma following health diagnoses (Osokpo & Riegel, 2021; Whitaker et al., 2024) may be important in how rehabilitation efforts are differentially experienced following cardiac events. Future research should more explicitly examine demographic influences, ideally with larger and more diverse samples.

Clinically, our findings are encouraging in that most patients benefited from cardiac rehabilitation regardless of psychological profile. However, considering groups differed significantly by baseline quality of life, relative differences persisted despite proportional gains. Importantly, approximately 30% of participants were categorized as ‘non‐adherent’, for whom no physical or psychological outcomes were able to be measured, and represent a concerning group with potentially unmet needs. Further research is therefore needed to understand the characteristics and needs of non‐completers, with additional research in early identification and tailored support for likely non‐completers.

## AUTHOR CONTRIBUTIONS

**Sean Hill:** Conceptualization; investigation; writing – original draft; methodology; writing – review and editing; formal analysis; project administration. **Sarah Scott:** Conceptualization; investigation; project administration; supervision. **Paul M. Salkovskis:** Conceptualization; investigation; methodology; writing – review and editing; formal analysis; project administration; data curation; supervision.

## FUNDING INFORMATION

No funding was received for this research.

## ETHICS STATEMENT

Ethics approval for this study was obtained following a favourable opinion from the Tyne and Wear South Research Ethics Committee (24/NE/0041). The study was sponsored by the University of Oxford Research Governance, Ethics & Assurance (RGEA, PID17286‐SI001), and approved via the Integrated Research Application System (IRAS ID 331618).

## Supporting information


Table S1.

## Data Availability

The data that support the findings of this study are available from the corresponding author upon reasonable request.
