# Supplementary material for: Health anxiety, mental defeat and fear of illness recurrence and progression following a cardiac event: Implications for cardiac rehabilitation
Source: Br J Health Psychol. 2026 Apr 17;31(2):e70068. doi: 10.1111/bjhp.70068 (PMC13088750; doi:10.1111/bjhp.70068)
Supplement: Supplementary file 1 — Table S1. [file BJHP-31-0-s001.docx]

**Supplementary document**

### **1. Full sample demographics**

**Table S1.**

*Full sample demographics.*

| **Measure** | **Whole Sample (N=176)** | **Main sample (N – [HA-only]; n=159)** | **HA+AD group (n=54)** | **AD-only group (n=29)** | **Non-clinical group (n=76)** | **HA-only (n=17)** | **Between-groups comparative statistics for whole sample (N=176)** |
| --- | --- | --- | --- | --- | --- | --- | --- |
| *Gender: n(%)* |  |  |  |  |  |  |  |
| Female | 46 (26.1) | 42 (26.4) | 22 (40.7) | 9 (31.0) | 11 (14.5) | 4 (23.5) | ***χ²* _(3, N=176)_=11.74, *p*=.008*; Cramer’s *V*=.258** |
| Male | 130 (73.9) | 117 (73.6) | 32 (59.3) | 20 (69.0) | 65 (85.5) | 13 (76.5) |  |
| *Ethnicity: n(%)* |  |  |  |  |  |  |  |
| White British | 130 (73.9) | 118 (75.6) | 37 (71.2) | 21 (72.4) | 60 (80.0) | 12 (70.6) | *χ²* _(12, N=172)_=15.60, *p*=.202; Cramer’s *V*=.174 |
| White Other | 20 (11.4) | 18 (11.5) | 7 (13.5) | 4 (13.8) | 7 (9.3) | 2 (11.8) |  |
| Asian (Mixed or Other) | 5 (2.8) | 12 (7.7) | 7 (13.5) | 4 (13.8) | 1 (1.3) | 1 (5.9) |  |
| Black African | 4 (2.3) | 3 (1.9) | 0 (0.0) | 0 (0.0) | 3 (4.0) | 1 (5.9) |  |
| Other Mixed | 4 (2.3) | 5 (3.2) | 1 (1.9) | 0 (0.0) | 4 (5.3) | 0 (0.0) |  |
| *Employment status: n(%)* |  |  |  |  |  |  |  |
| Retired | 73 (41.5) | 63 (39.6) | 22 (40.7) | 10 (34.5) | 31 (40.8) | 10 (58.8) | *χ²* _(12, N=176)_=9.09, *p*=.696; Cramer’s *V*=.131 |
| Full-time employed | 51 (29.0) | 48 (30.2) | 15 (27.8) | 12 (41.4) | 21 (27.6) | 3 (17.6) |  |
| Part-time employed | 27 (15.3) | 44 (27.7) | 17 (31.5) | 6 (20.7) | 21 (27.6) | 3 (17.6) |  |
| Unemployed | 4 (2.3) | 3 (1.9) | 0 (0.0) | 1 (3.4) | 2 (2.6) | 1 (5.9) |  |
| Student | 1 (0.6) | 1 (0.6) | 0 (0.0) | 0 (0.0) | 1 (1.3) | 0 (0.0) |  |
| *Marital status: n(%)* |  |  |  |  |  |  |  |
| Married | 136 (77.3) | 123 (77.4) | 44 (81.5) | 23 (79.3) | 56 (73.7) | 13 (76.5) | *χ²* _(12, N=176)_=5.05, *p*=.969; Cramer’s *V*=.098 |
| Partnered | 11 (6.3) | 10 (6.3) | 4 (7.4) | 1 (3.4) | 5 (6.6) | 1 (5.9) |  |
| Separated | 5 (2.8) | 5 (3.1) | 1 (1.9) | 2 (6.9) | 2 (2.6) | 0 (0.0) |  |
| Widowed | 9 (5.1) | 8 (5.0) | 2 (3.7) | 1 (3.4) | 5 (6.6) | 1 (5.9) |  |
| Single | 16 (8.5) | 13 (8.2) | 3 (5.6) | 2 (6.9) | 8 (10.5) | 2 (11.8) |  |
| *Smoking status: n(%)* |  |  |  |  |  |  |  |
| Smoker | 28 (15.9) | 26 (16.4) | 6 (11.1) | 6 (20.7) | 14 (18.4) | 2 (11.8) | *χ²* _(3, N=176)_=2.00, *p*=.574; Cramer’s *V*=.107 |
| Non-smoker | 148 (84.1) | 133 (83.6) | 48 (88.9) | 23 (79.3) | 62 (81.6) | 15 (88.2) |  |
|  |  |  |  |  |  |  |  |
| *Age: M (SD)* | 66.1 (10.0) | 65.6 (9.8) | 64.9 (10.0) | 66.2 (7.5) | 65.8 (10.6) | 70.6 (10.8) | *F*_(3,172)_=1.43, *p*=.24, *η²*=.02 |
| *BMI: M(SD)* | 28.1 (4.4) | 28.2 (4.3) | 28.1 (4.0) | 29.5 (5.1) | 27.7 (4.2) | 27.4 (4.7) | *F*_(3,171)_=1.45, *p*=.23, *η²*=.03 |
| *Days from event to contact: M(SD)* | 48.2 (24.7) | 47.7 (24.5) | 52.7 (26.4) | 42.7 (26.8) | 46.1 (21.6) | 52.7 (26.9) | *F*_(3,172)_=1.47, *p*=.23, *η²*=.03 |
|  |  |  |  |  |  |  |  |
| *Baseline depression* | 5.82 (4.6) | 6.2 (4.7) | 9.7 (4.0) | 8.2 (3.8) | 3.0 (2.8) | 2.2 (2.0) | ***F*_(3, 172)_=53.13, *p*<.001*, *η²*=.48** |
| *Baseline anxiety* | 6.94 (5.0) | 7.4 (5.0) | 10.8 (3.4) | 11.7 (3.3) | 3.3 (2.9) | 3.0 (2.4) | ***F*_(3, 172)_=94.90, *p*<.001*, *η²*=.62** |
| *Baseline health anxiety* | 12.21 (7.1) | 11.4 (6.9) | 19.5 (4.2) | 7.0 (4.3) | 7.4 (3.1) | 19.5 (4.9) | ***F*_(3, 172)_=141.56, *p*<.001*, *η²*=.71** |
| *Baseline quality of life* | 19.0 (6.0) | 19.0 (6.2) | 20.4 (5.7) | 21.6 (6.3) | 17.1 (6.1) | 18.7 (3.9) | ***F*_(3, 172)_=5.62, *p*=.001*, *η²*=.09** |
| *Statistically significant at least the *a*=.05 level, highlighted in bold for legibility.  ^1^ For comparative analyses, employment, marital, and ethnic status categories were collapsed due to small subgroup sizes. For employment, “full-time” and “student” were combined, “part-time” was not combined, and “retired” and “unemployed” were combined, to provide “full-time, part-time and not working”. For marital status, “married” and “partnered” were combined, as were “single,” “widowed,” and “separated,”, to provide “partnered or not partnered”. Ethnic status was combined to “White British” and all others to provide “Not White British”. These combinations were made to ensure sufficient cell counts for valid statistical comparison. | | | | | | | |
